# Screening of *LEP* gene polymorphisms as a risk factor for obesity and type 2 diabetes in Iraqis

**DOI:** 10.22099/mbrc.2019.34274.1423

**Published:** 2019-12

**Authors:** Maysoon Khudheyer Almyah, Adnan Issa Albadran

**Affiliations:** Department of Biology, College of Science, University of Basrah, Basrah, Iraq

**Keywords:** Screening, Obesity, Type 2 diabetes, Leptin, ob gene, Iraq

## Abstract

The prevalence of obesity and diabetes changes dramatically with lifestyle and unequal risk among individuals have made scientists interested to understand how the environment interferes with genetic factors to make it so-called genetic predisposition. This study aimed to explore wherethe most variable region is in leptin gene and analyse microsatellite repeats with direct sequencing in Iraqis and compare our alleles with other populations as a risk for obesity and T2D predisposition. DNA was extracted from blood of 60 type 2 diabetics and 70 non diabetics individuals, *LEP *5‛UTR, exon 2 and 3 were screened in 45 individuals (24 type 2 diabetes patients and 21 non- diabetics), *LEP* TTTC repeats region were amplified in all 130 participants from which 22 control samples were purified and sequenced, superimposed sequences were analyzed manually. Sequencing results showed G>A polymorphism (rs2167270) in 5‛UTR region. No polymorphisms detected in *LEP* exons 2 and 3. *LEP* microsatellites alleles were classified depending on sizes into class1 < (220bp) and class2 (> 220bp). Analysis of 22 control samples sequences of microsatellite region resulted in 6 type1 allele (unique sequence) and 5 type 3 allele (13 different isoforms) depending on TTTC arrangement separated by Ts bases. We concluded that *LEP* variations were in non- coding regions and no significant difference was observed in allele frequency between both groups, but there was a huge diversity in microsatellite repeat number and context among individuals. This may affects gene function thus prepare a predisposition for obesity and type 2 diabetes.

## INTRODUCTION

The terrible spread of metabolic disorders which is no longer confined to specific age group, has become a major problem facing the whole world, among them type 2 diabetes and obesity are the most common and linked diseases [1, 2]. Number of type 2 diabetic patients is in continuous increase because of changing life style in addition to genetic factors [3, 4]. Obesity is the fifth leading cause of death in the world and a main cause for other conditions e.g hypertention, corony heart disease, and diabetes [5]. Despite the prevalence of these non-infectious epidemics around the world, some individuals or families are almost excluded others are not, which simply explained by genetic predisposition [6]. 

**Figure 1:**
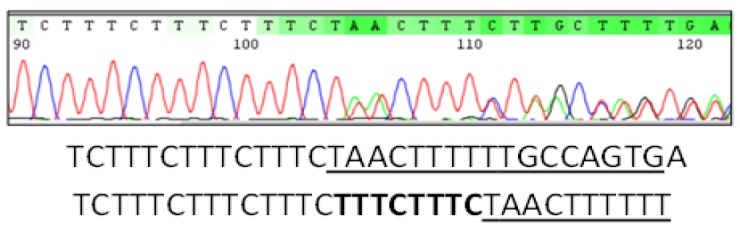
Mixed sequence traces showing microsatellite repeats (TTTC)n and underlined downstream sequence. This figure represents heterozygous genotype which includes two alleles. Analysis of this sequence showed that one allele had additional two repeats (**bold**) than other.

Leptin hormone is secreted from white adipose tissue and supposed to bind to its receptors in the hypothalamus in the brain to stop eating and regulate body fats in addition to insulin sensitivity [7, 8]; so leptin polymorphisms is a key factor linking obesity and type 2 diabetes [9]. *LEP* is discovered for the first time in mouse by Zhang and colleagues which is located on chromosom7q31.3, consists of 3 exons and two introns and its length is about 18 kb [10] (Suppliment Fig. 1). Microsatellite region consists of tetra nucleotides repeats (TTTC)n located in 476bp 3‛ of exon 3 which is a very variable region among ethnicities [11]. Regulatory regions of the gene such as 5‛UTR and 3‛UTR modulate gene expression [12] so inheritance of such variations plays an important role in the susceptibility to complex conditions such as obesity [13]. Different pathogenic mutations have been discovered in *LEP* for its role in obesity [14] like A19G variation in 5‛UTR region and G-2548A in promoter region [15, 16]. The effect of *LEP* polymorphisms on individuals’ phenotype differs among populations [17-20].

## MATERIALS AND METHODS

Sixty blood samples were collected from type 2 diabetic patients who were visiting Almouani and Alfaihaa’ hospitals, and other 70 non-diabetics volunteers from Basra population. All participants answered the questionnaire forma and gave their written agreement to participate in this study. BMI was calculated by dividing weight (in kilograms) by height (in meters) squared (kg/m2). 

Genomic DNA was extracted from peripheral blood using the Geneius Micro gDNA Extraction Kit (Geneaid, Korea) following the manufacturer’s instructions. *LEP* microsatellite region were detected in all 130 participants, but LEP exons were screened in 45 samples only (24 type 2 diabetes patients and 21 non diabetics) because the same samples were subjected for screening of *LEPR* promoter and 18 coding exons which was costly and time consuming [21]. PCR reactions were 2µl of each gDNA (70 ng), 0.025 µM of each primer (Spplement Table 1) added to lyophilized AccuPower PCR Premix (Bioneer, Korea) and the volumes were adjusted to 50 µl with deionized distilled water. PCR programs were: 95°C for 5 minutes, followed by 30 cycles of 95°C for 30 seconds; 60°C for amplification of exons 1, 2 and microsatellite region (63°C for 5‛UTR) for 30 seconds, 72°C for 30 seconds, and the final extension was 72°C for 5 minutes. We designed microsatellite primers because of low amplification efficiency of previous described primers [22]. PCR products were separated by 2% agarose gel electrophoresis stained with ethidium bromide. To detect *LEP* microsatellite repeats context, 22 samples from control group were sequenced and analyzed precisely. 

*LEP* microsatellite alleles were classified according to their sizes into class1 (<220bp) and class 2 (>220bp). In order to determine exact allele size for heterozygous, we purified each band separately from gel by Gel/PCR DNA fragment extraction kit (Genaid, Taiwan). All purified samples were amplified and run on agarose gel again using the same first PCR and electrophoresis conditions to be sure from the purity of bands. All amplified *LEP* 5‛UTR, exon2, exon3, homozygous and re-amplified purified heterozygous microsatellite fragments have been sequenced (Macrogen, Seol, Korea). 

After receiving sequencing results, a number of microsatellite sequences were heterozygous which appeared as mixed traces in chromatograms. This made the detection of alleles very complicated. Decoding of superimposed traces was done according to Dmitriev and colleagues [24] as in Figure 4. After resolving all resulted sequences, each of class1 and class 2 alleles were classified according to repeats arrangement context as described by Moffett and colleagues [11]. Odd ratios were calculated online (available at https://www.medcalc. org/calc/odds _ratio.php), P<0.05 were considered as significant. Means were calculated with ± standard deviation.

## RESULTS

Since obesity and diabetes have become a real problem by changing lifestyle, also obesity itself is a risk factor for diabetes and other complications, therefore we targeted 5‛UTR, coding exons and microsatellite region in obesity related gene (*ob*). Amplification of microsatellite region showed different sizes classified into class 1 (<220bp) and class 2 (>220bp) (Supplement Fig. 2). Sequencing results of *LEP* 5′UTR and exon 1 showed G>A polymorphism (rs2167270) in 5′UTR region with three genotypes (Suppement Fig. 3). No polymorphisms were detected in *LEP *exons 2 and 3. *LEP* microsatellite alleles were classified into class 1 (< 220bp) and class 2 (>220bp) depending on sizes in comparison to 100bp marker. 

Table 1 demonstrates different parameters, type 2 diabetics had significant higher BMI and waist circumference than other group. No significant difference was onserved in *LEP *alleles distribution between both groups (Table 2). We sent 22 control samples for direct sequencing from non-diabetics group. Decoding and analysis of mixed sequences traces has done manually (Fig. 1). 

**Table 1:** Different parameters in both groups

**parameters**	**Non-diabetes (70)**	**Diabetes (60)**
Age: range Average ± SD	30-75 48.1±9.2	30-80 49.8±11.4
Sex: male Female	11 59	9 51
BMI	30.0±7	33.3±5.6
WC	95.2±13.2	108.4±11.9

**Table 2:** Distribution of *LEP* polymorphisms in T2D and non-diabetics

**Genotype**	**Non-diabetics n (%)**	**Diabetics n (%)**	**OR**	**95% CI**
**5‛UTR (n=45)**				
GG	8 (38.1)	7 (29.2)	Ref.	
AA	2 (9.5)	4 (16.6)	2.3	0.41
GA	11 (52.4)	13 (54.2)	1.4	0.65
**Microsatellites (n=130)**				
Class1	10 (14.3)	12(20)	Ref.	
Class2	22 (31.4)	15(25)	0.57	0.30
Hetero	38(54.3)	33(55)	0.72	0.66

Class I individuals showed 6 alleles with 11, 12,13,14,15, and 18 repeats arranged in a common context demonstrated in Table 3. We have got 18 different genotypes from total 22 sequenced control samples, a high heterogeneity appeared in our results not only at class level but also in the same class (Table 4). Class 2 was very complex, 13 polymorphisms were formed from 5 alleles with 28, 29, 30, 31, and 34 repeats. For example, allele with 28 repeats has also 3 forms differ in the first inserted T position after repeat 12, 14, and 15 in the three isoforms, respectively, which separates the repeats into two parts, but all of them have the same repeats number (TTTC)_25_ followed by TT insertion before last three repeats. Only 34 repeats allele was in one form (Table 4).

Elbaz and colleagues have demonstrated in their study a comparison among world populations regarding distribution of class 1 and class 2 alleles [25], so we inserted our results in Table 5, Iraq was very close to Taiwan, Hong Kong, and Malaysia and has a high number of alleles when we take in consideration few sequenced samples. All decoded sequences were submitted to NCBI and DDBJ as Iraqi *LEP* microsatellite alleles, the accession numbers are: MH497009.1, LC415437, LC415438, LC415439, LC415440, LC415441, LC415442, LC415443, LC415444, LC415445, LC415446, LC415447, LC415448, LC415449, LC415450. LC431600, LC431601, LC431602, LC431603 and LC431604.

**Table 3:** Distribution of alleles depending on repeats number

**No. of TTTC**	**Repeats forms**	**Frequency**
**Class 1**	xxx(TTTC)nTyyy	
11	TTTC11	2
12	TTTC12	6
13	TTTC13	6
14	TTTC14	2
15	TTTC15	1
18	TTTC18	1
		
**Class 2**	Xxx(TTTC)nT(TTTC)mTT(TTTC)3TTyyy	
28	TTTC_12_TTTTC13TTTTTC3 TTTC_14_TTTTC11TTTTTC3 TTTC15TTTTC10TTTTTC3	6
29	TTTC12TTTTC14TTTTTC3 TTTC13TTTTC13TTTTTC3 TTTC15TTTTC11TTTTTC3	3
30	TTTC12TTTTC15TTTTTC3 TTTC13TTTTC14TTTTTC3 TTTC14TTTTC13TTTTTC3	5
31	TTTC12TTTTC16TTTTTC3 TTTC13TTTTC15TTTTTC3 TTTC15TTTTC13TTTTTC3	7
34	TTTC13TTTTC18TTTTTC3	1

**Table 4:** Distribution of individuals’ genotypes

**Genotypes**	**Repeats forms**	**No. of individuals**
11,13	TTTC11, TTTC13	1
11,14	TTTC11, TTTC14	1
12,12	TTTC12, TTTC12	2
12, 30	TTTC12, TTTC14TTTTC13TTTTTC3	2
13,13	TTTC13, TTTC13	1
13,15	TTTC13, TTTC15	1
13,18	TTTC13, TTTC18	1
13,28	TTTC13, TTTC12TTTTC13TTTTTC3	2
14,28	TTTC14, TTTC12TTTTC13TTTTTC3	2
28,30	TTTC12TTTTC13TTTTTC3, TTTC13TTTTC14TTTTTC3	1
28,31	TTTC12TTTTC13TTTTTC3, TTTC12TTTTC16TTTTTC3	1
28,31	TTTC14TTTTC11TTTTTC3, TTTC13TTTTC15TTTTTC3	1
28,34	TTTC12TTTTC13TTTTTC3, TTTC13TTTTC18TTTTTC3	1
29,29	TTTC12TTTTC14TTTTTC3, TTTC12TTTTC14TTTTTC3	1
29,30	TTTC12TTTTC14TTTTTC3, TTTC14TTTTC13TTTTTC3	1
30,31	TTTC12TTTTC15TTTTTC3, TTTC13TTTTC15TTTTTC3	1
31,32	TTTC13TTTTC15TTTTTC3, TTTC14TTTTC15TTTTTC3	1
31,34	TTTC15TTTTC13TTTTTC3, TTTC13TTTTC18TTTTTC3	1

## DISCUSSION

After 2003, improved economic conditions and entrance of modern technology was followed by unhealthy behaviors e.g. low activity, insufficient sleeping and high calories diets. Obesity has become a serious problem involving all ages and a risk factor for other complications such as high blood pressure, lipids, heart disease, and type 2 diabetes [5]. Despite of all that, some individuals still rationally are protected from these diseases likely due to genetic components of those individuals family history. Therefore, we targeted *ob* or *LEP* gene which linked with appetite disorder and insulin dis-regulation [28]. 

**Table 5:** Comparison of class 1 and class 2 alleles’ distribution in Iraqis and other populations in addition to number of yielded alleles

**Populations**	**Number of alleles in normal**	**class1% / class2%**	
Indian	13	61/39	[26]
Costa Ricans	11	58/42	[11]
Cyprus	-	69/31	[11]
Madagascar	22	89/11	[11]
Russians and British	11	82/18 and 80/15	[11]
Hong Kong	18	30/70	[11]
Taiwanese	12	23/77	[11]
Malaysians	12	39/61	[11]
Central Africans	14	93/07	[11]
West Africans	17	95/05	[11]
Euro Americans	14	49/51	[27]
Samoans	9	13/87	[27]
African Americans	19	91/07	[27]
Egyptians	5	97/03	[25]
Iraqis	19	38/62	This study

*LEP* A>G polymorphism known as A19G in 5′UTR variation was detected among Iraqi population in this study. Although this *LEP* SNP located in non-coding exon, but it was found to be associated with severe obesity and increase leptin levels due to its critical position in 5′UTR regulatory region [29]. No polymorphisms detected in exons 2 and 3. In 1996, Shintani and colleagues had discovered an extreme variable microsatellite region in *LEP*, 3912bp 3′ of stop codon. They found that this locus is a good marker for association studies of *LEP* with metabolic diseases such as obesity and type 2 diabetes [22]. Neel, 1962 explained genetic predisposition of obesity and diabetes as theory of thrifty genes [30]. We supposed?? that there is activation and positive selection of genes responsible for storage of fat as large as possible to be used at the time of starvation. Therefore these genes became common and wide spread in populations. But nowadays, with high calorie diets, previously useful genes have become risk factor for obesity and diabetes. To better understanding the evolutionary development of *LEP* gene repeats units variability among populations, Moffett and colleagues genotyped this locus in 1,957 individuals from 12 world populations [11], in addition to classify *LEP* gene according to size into two class, each class repeats were arranged in different context to form 4 types, type 1 is common among populations, type 2 constricted in African derived individuals, divided into 2a which is similar to type 1 except one base change and 2b which differ in its sequence from other types and is similar to *Pan *species. They explained that as genetic convergent between human and apes. In Iraqi population, we found only two types, the common short type 1 alleles which have a known structure xxx(TTTC)nTyyy, including 11, 12, 13, 14, 15, and18 repeats as well as other populations. The other type is previously described as type 3 which is more complicated than type 1 due to different arrangement of repeats separated by Ts. This type is abundant in Asian and European populations. Scientists have tried to explain this great heterogeneity in the microsatellite; Payseur and Nachman have proven that this heterogeneity is not linked to genetic recombination rate [31]. It is obvious in our study that the rate of heterogeneity in this site is related to different races. 

**Figure 2:**
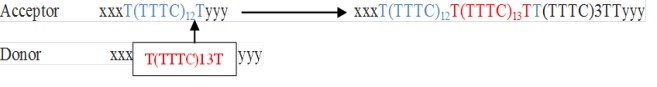
Theoretical explanation of type 3 allele’s evolvement from type1 during genetic recombination process, both acceptor and donor are type1 alleles, deletion of repeats region and subsequently deteriorated by natural selection. Presence of TTTC3 in the end of repeats is an evidence of a very short allele, gradually disappeared or merged with other forms.

It is possible to observe the linkage between genetic recombination among peoples and *LEP* microsatellite heterogeneity. Theoretically, we hypothesized that this broad genetic recombination has occurred since ancient times when mating was random, and showed rather stability in zones where mating has become restricted, but continued in others, which explains why they have other genetic patterns as Africans who are distinguished by having different allele (type 2a) other than common type 1 among world populations and type 3 which is found abundantly among Asians [11]. Moffett and colleagues believed that the type 3 did not arise from simple extending of type 1 allele, in an attempt to understand the evolvement of *LEP* gene polymorphisms in this region [11]. But when we look at the pattern of the type 3 alleles, we can clearly observe that the number of first two segments repeats separated by the base T is quite similar to type 1 sequence (Fig. 2). This can be theoretically explained as a genetic recombination during chromosomal crossover which resulted in deletion of this region in one of the two non-sister chromatids of homologous chromosomes and insertion in other.

## Supplementary Materials

Supplement
