# Supplementary material for: Screening of LEP gene polymorphisms as a risk factor for obesity and type 2 diabetes in Iraqis
Source: Mol Biol Res Commun. 2019 Dec;8(4):156–65. doi: 10.22099/mbrc.2019.34274.1423 (PMC6995335; doi:10.22099/mbrc.2019.34274.1423)
Supplement: Supplement [file mbrc-8-159-s001.pdf]

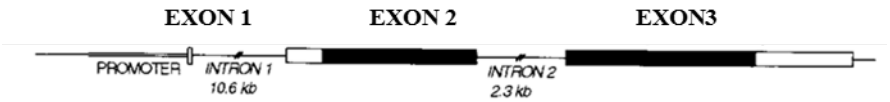

Figure S1: Schematic figure represents *LEP* gene regions, promoter represented by double line followed by untranselated exon 1. Exons 2 and 3 are the coding regions (black boxes) [23].

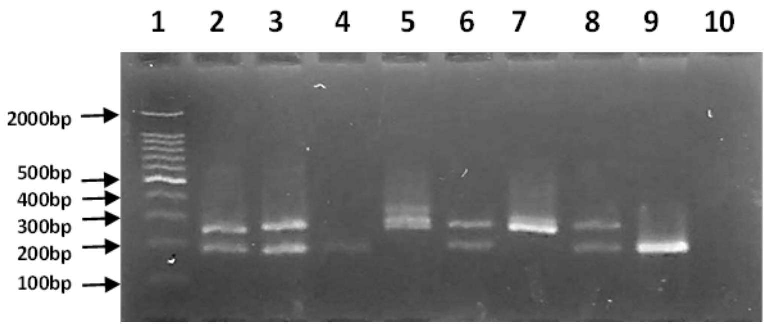

Figure S2: Agaros gel electrophoresis of *LEP* gene microsatellite alleles, lane 1: 100bp marker. Lanes 5 and 7 are class 2. Lanes 2, 3, 6 and 8 are class 1/class 2. Lane 10 is negative control.

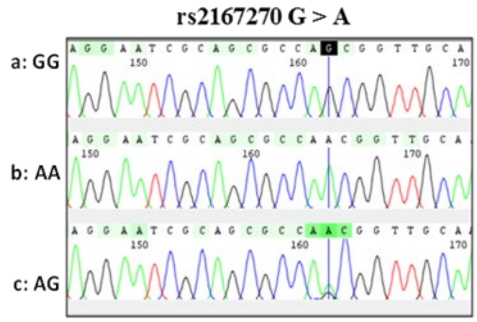

Figure S3: *LEP* 5'UTR genotypes, a: homo for G allele; b: homo for A allele and c: heterozygous (GA).

Table S1: Primers used in screening of *LEP* gene.

***LEP* Primers**	**Target**	**Amplicon size bp**	**Reference**
forward 5′-GCCCCGCGAGGTGCACACTG-3	5′UTR	213	[23]
Reverse 5′-GAGCGCGCCGGGGCCTTAC-3′			
forward 5′-TCTGTTTTCAGGCCCAAGAA-3′	Exon 2	203	[17]
Reverse 5′-TCCCCGGATACTCTCCTTA-3′			
forward 5-TCCCTCTTCCTCCTGCATA-3′	Exon 3	404	[17]
Reverse 5′-GCAGGAAGAGTGACCTTCAA-3′			
forward 5′-CCATTCTGAGTTCAAATAGAGGTCC-3′	Microsatellite	185-250	This study
Reverse 5′-TGGAATTGGGACACATGTTCTCA-3′			
